# Atrial Arrhythmias in Obstructive Sleep Apnea: Underlying Mechanisms and Implications in the Clinical Setting

**DOI:** 10.1155/2013/426758

**Published:** 2013-04-03

**Authors:** David Filgueiras-Rama, Miguel A. Arias, Ángel Iniesta, Eduardo Armada, José L. Merino, Rafael Peinado, J. L. López-Sendón

**Affiliations:** ^1^Cardiac Electrophysiology Unit, Department of Cardiology, Hospital Universitario La Paz, Paseo de la Castellana 261, 1st floor, 28046 Madrid, Spain; ^2^Robotic Cardiac Electrophysiology and Arrhythmia Unit, Hospital Universitario La Paz, Madrid, Spain; ^3^Cardiac Arrhythmia and Electrophysiology Unit, Department of Cardiology, Hospital Virgen de la Salud, Toledo, Spain; ^4^Acute Cardiac Care Unit, Department of Cardiology, Hospital Universitario La Paz, Madrid, Spain; ^5^Department of Cardiology, Hospital Universitario La Paz, Madrid, Spain

## Abstract

Obstructive sleep apnea (OSA) is a common disorder characterized by repetitive interruption of ventilation during sleep caused by recurrent upper airway collapse, which leads to intermittent hypoxia. The disorder is commonly undiagnosed despite its relationship with substantial cardiovascular morbidity and mortality. Moreover, the effects of the disorder appear to be particularly dangerous in young subjects. In the last decade, substantial clinical evidence has identified OSA as independent risk factor for both bradyarrhythmias and tachyarrhythmias. To date the mechanisms leading to such arrhythmias have not been completely understood. However, recent data from animal models and new molecular analyses have increased our knowledge of the field, which might lead to future improvement in current therapeutic strategies mainly based on continuous positive airway pressure. This paper aims at providing readers a brief and specific revision of current knowledge about the mechanisms underlying atrial arrhythmias in OSA and their clinical and therapeutic implications.

## 1. Introduction

Obstructive sleep apnea (OSA) is a relevant health problem because of its high prevalence and concomitant severe effects in the general population [1]. Although the disorder is mainly present in obese men, other clinical characteristics such as age 65 years or older, smoking, and alcohol consumption are also common [1]. OSA is characterized by repetitive interruption of ventilation during sleep caused by recurrent upper airway collapse, which is associated with increasing respiratory efforts and intermittent arterial oxygen desaturation. Nocturnal arousals, loud intermittent snoring, and increased daytime sleepiness are the main symptoms [2]. Obstructive apneas and hypopneas are considered significant if they last for more than 10 seconds. Such episodes can be recorded and quantified by a polysomnography study, which remains the standard technique for the diagnosis. Currently, international guidelines consider the diagnosis of OSA in the presence of an apnea-hypopnea index ≥5 and persistent respiratory effort during the episodes [2]. 

Cardiovascular morbidity and mortality significantly increase in patients with untreated severe OSA [3]. Moreover, the effects of the disorder appear to be particularly dangerous in young subjects [3, 4]. Substantial evidence supports the association between OSA and cardiovascular diseases, among which atrial arrhythmias and conduction disorders are of particular interest [5, 6]. Abnormalities such as hypoxia or sympathovagal imbalance are consistently present in OSA and have been associated with atrial arrhythmias [7, 8]. Moreover, under continuous positive airway pressure (CPAP) treatment, it has been shown the disappearance of both atrial fibrillation (AF) and heart block in patients with severe OSA [8, 9]. In this work we aim at reviewing the current knowledge about the mechanisms underlying atrial disorders in OSA and their implications in clinical practice, in such a way that clinicians will be able to establish a mechanistic link from molecular basis to animal models and clinical setting. 

## 2. Mechanisms of Increased Risk of Atrial Arrhythmias in OSA

**Figure 1:**
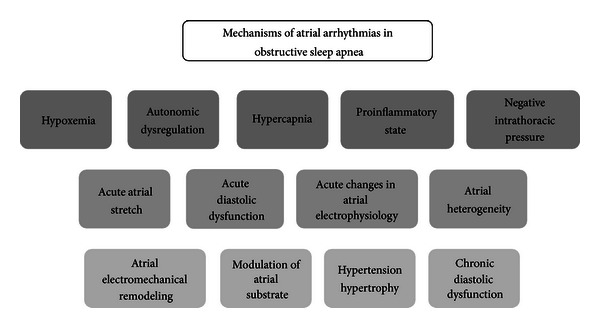
Diagram of mechanisms involved in atrial arrhythmias in patients with obstructive sleep apnea.

Different abnormalities have been involved in the pathogenesis of atrial arrhythmias and increased cardiovascular risk in patients with OSA [2]. Significantly relevant is the strong association between tachyarrhythmias, especially AF and OSA [5]. Thus hypoxia, hypercapnia, negative intrathoracic pressure, autonomic alterations, inflammation, increase in intravascular volume, and left ventricular diastolic dysfunction are likely implicated in a multifactorial process leading to functional and structural changes prompt to atrial arrhythmias (Figure 1). 

Hypoxemia significantly impairs relaxation of left and right ventricles in healthy humans rendered hypoxemic by breathing a variable nitrogen/oxygen mixture [10]. Similarly, intermittent apnea-induced hypoxemia documented during a polysomnographic study has been associated with significant hemodynamic changes in left and right ventricular functions [11, 12]. Thus, the severity of apnea-related hypoxemia is associated with a gradual deterioration of left ventricular filling, which may explain the presence of left ventricular hypertrophy in patients with OSA and no history of hypertension [13, 14]. Experimental evidence from animal models has yielded some clues about the molecular mechanisms leading to diastolic dysfunction. Thus, intermittent hypoxia seems to increase both oxidative stress and susceptibility of the heart to such a stress [15, 16]. In addition, myocyte hypertrophy, apoptosis, and multifocal infarcts have been also associated with left ventricular dysfunction [17]. Interestingly, hypoxia-induced deterioration of left ventricular filling is significantly correlated with acute atrial changes. In a rat model of obesity and acute OSA, increases in left ventricular end diastolic pressure during obstructive apnea correlated with significant left atrial enlargement monitored by echocardiography [18]. Moreover, in the same study Iwasaki et al. tried to elucidate the main mechanism leading to reproducibly inducible AF in those animals. To do so, they tested four different interventions consisting of pharmacologic autonomic blockade, ventilatory muscle paralysis, balloon occlusion of the inferior vena cava, and saline injection as control. Autonomic blockade partially decreased AF inducibility compared with control. However, only prevention of left atrial dilatation by balloon occlusion of the inferior vena cava was associated with significant suppression of AF inducibility [18]. The latter suggests that acute atrial dilatation due to apnea-related increase in left ventricular end diastolic pressure may prompt the atria to AF.

The results are especially relevant since acute atrial stretch has been traditionally associated with increased vulnerability to AF. Moreover, resumption of sinus rhythm is consistently achieved after releasing the atrial stretch [19]. The latter has important clinical implications in spite of the fact that mechanisms underlying stretch-induced AF are highly complex and not completely understood [20]. Therefore, AF might be terminated or prevented by correcting apnea-induced acute hemodynamic changes in the left atrium. 

In addition, hypoxia may affect atrial electrophysiology by modifying conduction velocity and atrial refractoriness. However, data from different animal models have yielded divergent results, which preclude identifying conclusive effects [21, 22]. High levels of hypercapnia may also affect atrial electrophysiology by slowing atrial conduction and increasing atrial refractoriness. Upon returning to normocapnia, refractoriness rapidly returns to baseline levels while conduction slowing still persists. Although it is difficult to reproduce *in vivo *responses to acute hypoxemia and hypercapnia, hypercapnia-related electrophysiology changes have been associated with AF in the sheep intact heart [23]. 

Autonomic neural inputs to ganglionated plexi occurring during apnea may initiate AF. However, the exact mechanisms leading to AF are extremely difficult to elucidate because of the anatomic and physiological complexity of the sympathetic and parasympathetic innervation of the heart [24]. Ganglionated plexi neural activity progressively increases before the onset of AF, which associates with shortening of the atrial refractory period [25]. The latter allows nonconducted premature stimuli at baseline to activate the atrium and probably generate heterogeneity [26], which leads to reentry and AF. Moreover, either the blockade of both arms of the autonomic nervous system or ablation of pericardiac fat pad containing ganglionated plexi inhibits the occurrence of apnea-induced AF, similar to the previously mentioned effect of releasing atrial stretch [25]. However, acute effects of autonomic blockade may not last in the long term, rendering AF reinducible after several weeks of ablation-based autonomic denervation [27]. It is difficult to determine whether autonomic inputs or atrial stretch plays the main role in initiating and sustaining AF. Model-based differences on the presence or absence of negative intrathoracic pressure during apnea might be responsible for more stretch-dependent or autonomic deregulation-dependent AF [18, 25]. 

Negative intrathoracic pressure occurs during OSA due to ineffective inspiratory efforts against the occluded upper airway. Animal models simulating clinical OSA incorporate negative intrathoracic pressure during tracheal occlusion, which leads to shortening of the atrial effective refractory period and action potential duration [18, 28]. Such electrophysiological changes increase AF susceptibility, which seems to be partially mediated by vagal activation. The latter is further supported by a high rate of AF prevention upon atropine or vagotomy [28]. Concomitant changes in sympathetic activity and atrial pressure have been also documented [18, 29], which may increase atrial susceptibility to AF in the presence of vagal activation. Acetylcholine-mediated AF is facilitated by isoproterenol, which decreases the threshold of acetylcholine concentration for AF induction and increases AF duration [30]. Similarly, increases in left atrial pressure and atrial dilatation decrease the conduction velocity and increase the degree of spatial heterogeneities in conduction, which facilitates reentry and AF [31]. Both phenomena, along with vagal activation may generate an optimal substrate to initiate and sustain AF. Additionally, in the presence of hypoxia it might be expected a significant role of the ATP-sensitive K^+^ current (*I*_K-ATP_). However, its role seems to be much less prominent and probably overcome by the vagal-mediated activity [28]. 

Inflammation may facilitate AF in patients with atrial substrate suitable to develop the arrhythmia. It is also possible a direct effect of inflammatory markers on ionic channels and signaling pathways involved in the development of atrial fibrosis, both leading to AF [32]. Interestingly, as inflammatory cytokines rise the risk of AF concomitantly increases [33]. Intermittent hypoxia and hypercapnia in animal models result in systemic inflammation and increases in interleukin-6 (IL-6) [34]. The mechanism by which IL-6 may be produced in hypoxic conditions is the upregulation of transcription factors NF-*κ*B (nuclear factor kappa-light-chain-enhancer of activated B cells) and NF-IL6 (nuclear factor for IL-6 expression) [35]. In addition, increases in IL-6 precede increases in C-reactive protein (CRP), which is an inflammatory mediator involved in releasing new proinflammatory cytokines. Patients with OSA show markedly elevated monocyte and neutrophil NF-*κ*B activity [36]. Therefore, a proinflammatory state may lead to higher susceptibility to AF in OSA patients. Moreover, inflammation is common in OSA patients, including elevated levels of proinflammatory cytokines. Tumor necrosis factor-*α* (TNF-*α*) and IL-6 levels are elevated in OSA independent of obesity [37, 38]. However, the initial trigger driving the elevation of proinflammatory cytokines in OSA patients has not been completely elucidated. Some evidence supports the role of both sides of the autonomic nervous system and hypoxia as the main initiation mechanisms [39].

Some of the mechanisms involved in tachyarrhythmias have been also associated with bradyarrhythmias or asystole in patients with severe OSA. In fact, bradycardia, significant pauses (>3 s) and some degree of conduction block are highly prevalent in OSA patients [40]. The occurrence and degree of bradycardia during apnea depends on the degree of hypoxemia resulting from these apneas. Furthermore, increased vagal efferent activity appears to cause the bradycardia, which can be prevented by intravenous atropine [41]. Paroxysmal parasympathetic discharges are more pronounced during rapid eye movement (REM) sleep, which even in healthy young subjects have been associated with marked bradycardia and prolonged asystolic pauses [42]. In patients with OSA marked bradycardia may occur particularly in REM state, during which longer breathing pauses and greater degrees of oxyhemoglobin desaturation occur. In addition, in OSA there is lack of normal lung expansion during ventilation, which prevents attenuation of parasympathetic discharges by vagolytic properties of normal lung expansion [43]. The influence of parasympathetic tone is further supported by clinical electrophysiological studies revealing no evidence for advanced sinus node disease or atrioventricular conduction system dysfunction, which suggest that prolonged ventricular asystole during OSA is not due to fixed or anatomic disease of the sinus node or the atrioventricular conduction system [44]. Animal models aimed at studying AF in OSA have also shown slowing of the heart rate concurrent with increased blood pressure after the initiation of apnea [25, 28]. The effects are compatible with simultaneous increases in cardiac parasympathetic and vasoconstricting sympathetic tone, similar to the diving reflex seen in mammals, during which the body responds to apnea by increasing sympathetic tone to the peripheral vasculature and parasympathetic tone to the heart [45]. 

## 3. Clinical Significance of Atrial Arrhythmias in OSA Patients

In the early 80's a potential relationship between OSA and AF emerged from an observational, uncontrolled study by Guilleminault et al., in which the use of 24-hour holter electrocardiography identified a prevalence of nocturnal paroxysms of AF of *≈*3% in OSA patients compared with the general population prevalence of 0.4 to 1% [46]. The same study reported sinus arrest lasting for more than 2.5 seconds and second-degree atrioventricular conduction block in 10% and 7% of OSA patients, respectively. 

In a large cohort of 3542 patients who underwent their first diagnostic polysomnogram study and further average follows up of 4.7 years, obesity and OSA were independent risk factors for incident AF in individuals <65 years of age [47]. Moreover, the magnitude of nocturnal oxygen desaturation is consistently present in several studies as independently predictive of AF [47–49]. The latter provides further support to hypoxemia-related pathophysiological changes in AF onset. 

OSA and AF have been linked in different clinical settings. Thus, an apnea-hypopnea index (AHI) ≥5 was associated with significant higher risk of postcoronary artery bypass surgery AF compared with lower values of AHI [49]. Recurrence of AF was also associated with untreated OSA in a prospective cohort of patients who underwent electrical cardioversion and one-year follow up [48]. More recently, data from several observational studies have supported the assertion that OSA patients diagnosed using polysomnography have significantly greater AF recurrence rates after pulmonary vein isolation compared with controls [50]. Such a higher risk of recurrences might be explained by significant differences in atrial remodeling compared with patients who underwent catheter ablation of AF and an AHI ≤15. In fact, OSA is associated with significant larger atrial enlargement, lower atrial voltage, and more site-specific and widespread conduction abnormalities [51]. 

OSA is also an independent risk factor for stroke. Therefore it would be particularly relevant to determine a potential increased risk of stroke in AF patients with OSA. Although such data is not available to date, some clues indicate that OSA may have a role in stroke risk stratification scores in patients with AF. Thus, OSA is strongly associated with AF and most of the relative risks included in current stroke risk stratification scores are very similar between AF and OSA patients [52, 53]. In addition, OSA is associated with other risk factors in AF such as hypertension and diabetes mellitus [54].

Bradyarrhythmia and sinus pauses are commonly described in patients with OSA. However, there is a huge variability between trials, which show incidences ranging from 5% to 50% [6, 40, 46]. Even no differences in conduction delay disturbances between severe sleep-disordered breathing and controls have been reported using retrospective ECG data recorded during the sleep period [55]. Noncomparable study designs, methods, and populations may explain such differences. The large intraindividual variability reported by Simantirakis et al. in a cohort of patients with moderate to severe OSA who underwent continuous monitoring by implantable loop recorder [40], demonstrates the incapability of 24-hour and 48-hour holter monitoring to accurately determine the incidence of atrial arrhythmias. Therefore, assuming that continuous ECG monitoring is the most reliable tool to determine the incidence of cardiac arrhythmias, approximately half of OSA patients evidence severe cardiac rhythm disturbances [40]. Moreover, the frequency and severity of apnea-related nocturnal bradyarrhythmias correlate with body mass index, AHI, and desaturation level during the sleep study [6, 40, 56]. 

Other ECG parameters such as QT interval are affected by the severity of the sleep apnea. Thus, QT corrected interval is increased in patients with moderate to severe OSA [57]. Moreover, QT corrected interval dispersion, defined as the difference between the maximum and minimum QT intervals, shows a strong positive correlation with the AHI [58]. Nocturnal prolonged cycle lengths can facilitate the occurrence of early afterdepolarizations and ventricular arrhythmias including torsades de pointes. Both increased QT interval and QT corrected interval dispersion are of interest in OSA individuals particularly sensitive to nocturnal heart rate pauses and QT prolongation such as patients treated with class III antiarrhythmic agents or with diuretics, along with subsets of patients with the long QT syndrome. Although several factors are involved, serious and potentially fatal arrhythmias may occur during sleep in patients with OSA, which is especially relevant between 10 p.m. and 6 a.m [59] (see Table 1 with supplementary information about risk of atrial arrhythmias in OSA).

## 4. Clinical Implications of Appropriate Treatment in OSA Patients with Atrial Arrhythmias

From the foregoing, we have discussed and provided substantial scientific support about the mechanisms and clinical impact of atrial arrhythmias in OSA patients. However, to date it is still difficult to determine the exact role of OSA treatment in preventing atrial arrhythmias. Current clinical consensus recommends that OSA should be treated with continuous positive airway pressure (CPAP) for ventilatory support as well as a tool for secondary prevention of cardiac problems [60]. It is not clear either whether other modes of ventilatory support such as bilevel pressure support (BPAP) offer any advantages over the conventional CPAP. Nowadays, BPAP should be reserved for patients with ventilatory failure [60].

Direct evidence of long-lasting apneic events (*≈*48 s) preceding AF onset has been rarely reported [9]. However, it suggests a causal association between OSA and AF. Furthermore, a period without apneas leads to spontaneous resumption to sinus rhythm. Appropriate CPAP therapy in OSA patients with AF who underwent electrical cardioversion significantly reduced AF recurrences after 12-month followup. AF recurred in only 42% of OSA patients effectively treated with CPAP, compared to 82% in untreated patients. Interestingly, recurrence rates were also significantly lower than in randomly selected controls without previous history of polysomnography, which suggests the presence of undiagnosed OSA among controls [48]. Recently, a large series by Abe et al. has shown that treatment with CPAP therapy significantly prevents OSA-associated paroxysmal AF in patients with moderate to severe nocturnal apneas [61]. Similar to previous series and considering the above-mentioned limitation of limited electrocardiographic recording period during polysomnography [46], CPAP therapy significantly eliminates sinus bradycardia and sinus pauses. Only a trend to significant differences before and after CPAP therapy was also present in episodes of second-and third-degree atrioventricular block [61]. The latter may be explained by the limited monitoring period and very low rate of second- and third- degree atrioventricular block, which precludes reaching statistical significance. 

Untreated OSA patients have higher recurrence rates after catheter ablation of AF. Such higher recurrence rates are also associated with the presence of more prevalent nonpulmonary veins triggers [62]. More recently Naruse et al. have suggested that an AHI of more than 10 has predictive value of AF recurrences after AF ablation. Moreover, CPAP therapy reduces the risk of recurrent AF after pulmonary vein isolation in patients with an AHI >5 [63]. AF recurrences were also reduced in the followup of patients with atrial flutter who underwent radiofrequency ablation of cavotricuspid isthmus (CTI) and proper CPAP therapy [64]. However, CPAP was only protective from AF recurrences when AF was not present prior to CTI ablation. 

The exact mechanisms for preventing AF recurrences by CPAP therapy are not understood. Beyond preventing upper airway obstruction, correcting hypoxemia, and decreasing the AHI, CPAP therapy seems to attenuate oxidative stress and systemic inflammation [65], which has been associated with both incident AF and AF recurrences after catheter ablation [66, 67]. 

The effects of CPAP therapy have been also studied in patients with OSA and bradyarrhythmia. Cornerstone series by Guilleminault et al. in the early 80's initially suggested that preventing upper airway obstruction by tracheostomy completely abolished conduction disturbances at 6 months of followup [46]. Case reports have also documented complete reversion of second-degree atrioventricular block after initiation of CPAP treatment [68]. During polysomnography the vast majority of the apnea-associated bradyarrhythmias occur during rapid eye movement sleep and significant desaturation of at least 4%. Both CPAP and BPAP drastically decrease the AHI and bradyarrhythmias [69]. More accurate followup using implantable loop recorders shows that the initiation of CPAP therapy tends to reduce the total number of the recorded episodes in the short term while in the long term bradycardia episodes are completely abolished [40]. CPAP therapy also has the capability to decrease the QT corrected interval dispersion present at baseline in patients with moderate to severe OSA [58]. The latter might have implications in preventing bradycardia-related early afterdepolarizations and ventricular arrhythmias leading to nocturnal sudden death (see Table 2 with supplementary information about effects of CPAP therapy on atrial arrhythmia outcomes in OSA patients).

However, one important limitation of CPAP therapy is the poor long-term acceptance of treatment. Approximately 40% of patients are no longer complaint with the treatment after 3 years [70]. However, it seems that patients with the most severe sleep apnea are more likely to be complaint. The most common reason for discontinuing is intolerance of the mask [71]. Moreover, mask leak is a major independent predictor of CPAP compliance; therefore reducing mask leak predicts good compliance with CPAP therapy [72]. 

Finally, based on the benefits of CPAP therapy and such a frequent undiagnosed disorder in the general population, screening for OSA in patients with both AF and atrial flutter appears to be a reasonable clinical strategy if either a clinical or catheter-based rhythm control strategy is pursued. It seems also reasonable to identify patients with apnea-related bradyarrhythmias, since appropriate treatment may completely abolish its presence. 

## 5. Conclusions and Future Directions

Atrial arrhythmias are highly prevalent in patients with moderate to severe obstructive sleep apnea, which has been identified as independent risk factor for both bradyarrhythmias and tachyarrhythmias. Hypoxia, hypercapnia, autonomic dysfunction, acute atrial stretch, negative intrathoracic pressure, and inflammation are some of the mechanisms leading to arrhythmia. However, complete understanding of such mechanisms is still ongoing and further research based on animal models is needed. CPAP therapy has demonstrated significant improvement in preventing and even abolishing atrial arrhythmias. However, it is necessary to improve and develop alternatives to conventional CPAP, which help to prevent current high rates of treatment discontinuation in the long-term.

## Figures and Tables

**Table 1:** Risk of atrial arrhythmias in obstructive sleep apnea.

Author, year	Design	Study population	Diagnostic method	*N* of patients	Cardiac monitoring	Results
Guilleminault et al., 1983 [46]	Uncontrolled	Severe OSA	PSG	400	24 h holter ECG	Nocturnal paroxysms of AF in *≈*3% of OSA patients Sinus arrest and AV block in 10% and 7% of OSA patients

Gami et al., 2007 [47]	Observational retrospective	Adults underwent a 1st PSG study. No AF at baseline	PSG	3542	N/A. Medical index diagnostic codes for AF	Obesity and OSA were independent risk factors for incident AF

Mooe et al., 1996 [49]	Observational prospective	Patients underwent CABG surgery	PSG	121	Prospective monitoring until discharge	AHI ≥ 5 was associated with significant higher risk of postsurgical AF

Ng et al., 2011 [50]	Meta-analysis (observational studies)	Patients underwent PVI	PSG/Berlin questionnaire	3995	24/48 h holter ECG Event monitor/recorder Telephone follow-up ECG	OSA led to greater AF recurrence rates after PVI

Simantirakis et al., 2004 [40]	Observational prospective	Moderate-severe OSA	PSG	23	Implantable loop recorder	Cardiac pauses >3 s and bradycardic episodes <40 bpm in 47% of patients

Becker, 1998 [6]	Observational prospective	Unselected OSA	PSG	239	24 h holter ECG	2nd- and 3rd-degree AV block and/or sinus arrest in *≈*7.5% of OSA patients

Hoffstein and Mateika, 1994 [56]	Observational prospective	Patients underwent PSG study	PSG	458	Single lead ECG during PSG	58% prevalence of arrhythmias in OSA patients

AF: atrial fibrillation; AHI: apnea/hypopnea index; AV block: atrioventricular block; CABG: coronary artery bypass graft; OSA: obstructive sleep apnea; PSG: polysomnography; PVI: pulmonary vein isolation.

**Table 2:** Effects of CPAP therapy on atrial arrhythmia outcomes in OSA patients.

Author, year	Design	Study population	Diagnostic method	*N* of patients	Cardiac monitoring	Results
Kanagala et al., 2003 [48]	Observational prospective	Patients underwent AF/AFL DC shock	PSG	121	Hospital records Phone interviews ECG Medical visits	AF recurrences in 42% of OSA treated with CPAP AF recurrences in 82% of untreated patients

Abe et al., 2010 [61]	Nonrandomized prospective	Adults underwent PSG study	PSG	1456	ECG monitoring during PSG	CPAP prevented AF, sinus bradycardia/pauses in patients with moderate to severe OSA

Naruse et al., 2013 [63]	Nonrandomized prospective	Patients underwent PVI isolation	PSG	153	12-lead ECG 24 h holter ECG Portable ECG monitoring	Untreated OSA showed higher recurrence of AF after ablation

Koehler et al., 1998 [69]	Nonrandomized prospective	OSA patients with bradyarrhythmia	PSG	16	ECG monitoring during PSG	CPAP and BPAP drastically decreased bradyarrhythmias

Simantirakis et al., 2004 [40]	Observational prospective	Moderate-severe OSA	PSG	23	Implantable loop recorder	Long-term CPAP therapy completely abolished bradycardia episodes

AF: atrial fibrillation; AFL: atrial flutter; CPAP: continuous positive airway pressure; OSA: obstructive sleep apnea; PSG: polysomnography; PVI: pulmonary vein isolation.

## References

[1] Young T, Palta M, Dempsey J, Skatrud J, Weber S, Badr S (1993). The occurrence of sleep-disordered breathing among middle-aged adults. *The New England Journal of Medicine*.

[2] Parati G, Lombardi C, Hedner J (2012). Position paper on the management of patients with obstructive sleep apnea and hypertension: joint recommendations by the European Society of Hypertension, by the European Respiratory Society and by the members of European COST (COoperation in Scientific and Technological research) ACTION B26 on obstructive sleep apnea. *Journal of Hypertension*.

[3] Marin JM, Carrizo SJ, Vicente E, Agusti AGN (2005). Long-term cardiovascular outcomes in men with obstructive sleep apnoea-hypopnoea with or without treatment with continuous positive airway pressure: an observational study. *The Lancet*.

[4] Lavie P, Lavie L, Herer P (2005). All-cause mortality in males with sleep apnoea syndrome: declining mortality rates with age. *European Respiratory Journal*.

[5] Gami AS, Pressman G, Caples SM (2004). Association of atrial fibrillation and obstructive sleep apnea. *Circulation*.

[6] Becker HF (1998). Heart block in patients with sleep apnoea. *Thorax*.

[7] Tkacova R, Rankin F, Fitzgerald FS, Floras JS, Bradley TD (1998). Effects of continuous positive airway pressure on obstructive sleep apnea and left ventricular afterload in patients with heart failure. *Circulation*.

[8] Roche F, Xuong ANT, Court-Fortune I (2003). Relationship among the severity of sleep apnea syndrome, cardiac arrhythmias, and autonomic imbalance. *Pacing and Clinical Electrophysiology*.

[9] Schulz R, Eisele HJ, Seeger W (2005). Nocturnal atrial fibrillation in a patient with obstructive sleep apnoea. *Thorax*.

[10] Cargill RI, Kiely DG, Lipworth BJ (1995). Adverse effects of hypoxaemia on diastolic filling in humans. *Clinical Science*.

[11] Yang SQ, Han LL, Dong XL (2012). Mal-effects of obstructive sleep apnea on the heart. *Sleep Breath*.

[12] Kraiczi H, Caidahl K, Samuelsson A, Peker Y, Hedner J (2001). Impairment of vascular endothelial function and left ventricular filling: association with the severity of apnea-induced hypoxemia during sleep. *Chest*.

[13] Hedner J, Ejnell H, Caidahl K (1990). Left ventricular hypertrophy independent of hypertension in patients with obstructive sleep apnoea. *Journal of Hypertension*.

[14] Arias MA, García-Río F, Alonso-Fernández A, Mediano O, Martínez I, Villamor J (2005). Obstructive sleep apnea syndrome affects left ventricular diastolic function: effects of nasal continuous positive airway pressure in men. *Circulation*.

[15] Park AM, Suzuki YJ (2007). Effects of intermittent hypoxia on oxidative stress-induced myocardial damage in mice. *Journal of Applied Physiology*.

[16] Hayashi T, Yamashita C, Matsumoto C (2008). Role of gp91phox-containing NADPH oxidase in left ventricular remodeling induced by intermittent hypoxic stress. *American Journal of Physiology*.

[17] Farré R, Montserrat JM, Navajas D (2008). Morbidity due to obstructive sleep apnea: insights from animal models. *Current Opinion in Pulmonary Medicine*.

[18] Iwasaki YK, Shi Y, Benito B (2012). Determinants of atrial fibrillation in an animal model of obesity and acute obstructive sleep apnea. *Heart Rhythm*.

[19] Ravelli F, Allessie M (1997). Effects of atrial dilatation on refractory period and vulnerability to atrial fibrillation in the isolated Langendorff-perfused rabbit heart. *Circulation*.

[20] Filgueiras-Rama D, Martins RP, Mironov S (2012). Chloroquine terminates stretch-induced atrial fibrillation more effectively than flecainide in the sheep heart. *Circulation*.

[21] Krause PC, Inoue H, Zipes DP (1989). Electrophysiologic alterations produced by hypoxia in the canine heart. *American Heart Journal*.

[22] Lammers WJEP, Kirchhof C, Bonke FIM, Allessie MA (1992). Vulnerability of rabbit atrium to reentry by hypoxia. Role of inhomogeneity in conduction and wavelength. *American Journal of Physiology*.

[23] Stevenson IH, Roberts-Thomson KC, Kistler PM (2010). Atrial electrophysiology is altered by acute hypercapnia but not hypoxemia: implications for promotion of atrial fibrillation in pulmonary disease and sleep apnea. *Heart Rhythm*.

[24] Puodziukynas A, Kazakevicius T, Vaitkevicius R (2012). Radiofrequency catheter ablation of pulmonary vein roots results in axonal degeneration of distal epicardial nerves. *Autonomic Neuroscience*.

[25] Ghias M, Scherlag BJ, Lu Z (2009). The role of ganglionated plexi in Apnea-related atrial fibrillation. *Journal of the American College of Cardiology*.

[26] Moe GK, Rheinboldt WC, Abildskov JA (1964). A computer model of atrial fibrillation. *American Heart Journal*.

[27] Oh S, Zhang Y, Bibevski S, Marrouche NF, Natale A, Mazgalev TN (2006). Vagal denervation and atrial fibrillation inducibility: epicardial fat pad ablation does not have long-term effects. *Heart Rhythm*.

[28] Linz D, Schotten U, Neuberger HR, Böhm M, Wirth K (2011). Negative tracheal pressure during obstructive respiratory events promotes atrial fibrillation by vagal activation. *Heart Rhythm*.

[29] Linz D, Mahfoud F, Schotten U (2012). Renal sympathetic denervation suppresses postapneic blood pressure rises and atrial fibrillation in a model for sleep apnea. *Hypertension*.

[30] Sharifov OF, Fedorov VV, Beloshapko GG, Glukhov AV, Yushmanova AV, Rosenshtraukh LV (2004). Roles of adrenergic and cholinergic stimulation in spontaneous atrial fibrillation in dogs. *Journal of the American College of Cardiology*.

[31] Eijsbouts SCM, Houben RPM, Blaauw Y, Schotten U, Allessie MA (2004). Synergistic action of atrial dilation and sodium channel blockade on conduction in rabbit atria. *Journal of Cardiovascular Electrophysiology*.

[32] Ramos-Mondragón R, Vega AV, Avila G (2011). Long-term modulation of Na^+^ and K^+^ channels by TGF-*β*1 in neonatal rat cardiac myocytes. *Pflugers Archiv European Journal of Physiology*.

[33] Richter B, Gwechenberger M, Socas A (2012). Markers of oxidative stress after ablation of atrial fibrillation are associated with inflammation, delivered radiofrequency energy and early recurrence of atrial fibrillation. *Clinical Research in Cardiology*.

[34] Tam CS, Wong M, Tam K, Aouad L, Waters KA (2007). The effect of acute intermittent hypercapnic hypoxia treatment on IL-6, TNF-*α*, and CRP levels in piglets. *Sleep*.

[35] Matsui H, Ihara Y, Fujio Y (1999). Induction of interleukin (IL)-6 by hypoxia is mediated by nuclear factor (NF)-*κ*B and NF-IL6 in cardiac myocytes. *Cardiovascular Research*.

[36] Htoo AK, Greenberg H, Tongia S (2006). Activation of nuclear factor kB in obstructive sleep apnea: a pathway leading to systemic inflammation. *Sleep and Breathing*.

[37] Arias MA, García-Río F, Alonso-Fernández A (2008). CPAP decreases plasma levels of soluble tumour necrosis factor-*α* receptor 1 in obstructive sleep apnoea. *European Respiratory Journal*.

[38] Vgontzas AN, Papanicolaou DA, Bixler EO, Kales A, Tyson K, Chrousos GP (1997). Elevation of plasma cytokines in disorders of excessive daytime sleepiness: role of sleep disturbance and obesity. *Journal of Clinical Endocrinology and Metabolism*.

[39] Mills PJ, Dimsdale JE (2004). Sleep apnea: a model for studying cytokines, sleep, and sleep disruption. *Brain, Behavior, and Immunity*.

[40] Simantirakis EN, Schiza SI, Marketou ME (2004). Severe bradyarrhythmias in patients with sleep apnoea: the effect of continuous positive airway pressure treatment: a long-term evaluation using an insertable loop recorder. *European Heart Journal*.

[41] Zwillich C, Devlin T, White D (1982). Bradycardia during sleep apnea. Characteristics and mechanism. *Journal of Clinical Investigation*.

[42] Guilleminault C, Pool P, Motta J, Gillis AM (1984). Sinus arrest during REM sleep in young adults. *The New England Journal of Medicine*.

[43] Somers VK, Dyken ME, Skinner JL (1993). Autonomic and hemodynamic responses and interactions during the Mueller maneuver in humans. *Journal of the Autonomic Nervous System*.

[44] Grimm W, Hoffmann J, Menz V (1996). Electrophysiologic evaluation of sinus node function and atrioventricular conduction in patients with prolonged ventricular asystole during obstructive sleep apnea. *American Journal of Cardiology*.

[45] Fagius J, Sundlof G (1986). The diving response in man: effects on sympathetic activity in muscle and skin nerve fascicles. *Journal of Physiology*.

[46] Guilleminault C, Connolly SJ, Winkle RA (1983). Cardiac arrhythmia and conduction disturbances during sleep in 400 patients with sleep apnea syndrome. *American Journal of Cardiology*.

[47] Gami AS, Hodge DO, Herges RM (2007). Obstructive sleep apnea, obesity, and the risk of incident atrial fibrillation. *Journal of the American College of Cardiology*.

[48] Kanagala R, Murali NS, Friedman PA (2003). Obstructive sleep apnea and the recurrence of atrial fibrillation. *Circulation*.

[49] Mooe T, Gullsby S, Rabben T, Eriksson P (1996). Sleep-disordered breathing: a novel predictor of atrial fibrillation after coronary artery bypass surgery. *Coronary Artery Disease*.

[50] Ng CY, Liu T, Shehata M, Stevens S, Chugh SS, Wang X (2011). Meta-analysis of obstructive sleep apnea as predictor of atrial fibrillation recurrence after catheter ablation. *American Journal of Cardiology*.

[51] Dimitri H, Ng M, Brooks AG (2012). Atrial remodeling in obstructive sleep apnea: implications for atrial fibrillation. *Heart Rhythm*.

[52] Hart RG, Pearce LA, Albers GW (2007). Independent predictors of stroke in patients with atrial fibrillation: a systematic review. *Neurology*.

[53] Valham F, Mooe T, Rabben T, Stenlund H, Wiklund U, Franklin KA (2008). Increased risk of stroke in patients with coronary artery disease and sleep apnea: a 10-year follow-up. *Circulation*.

[54] McNicholas WT, Bonsignore MR (2007). Sleep apnoea as an independent risk for cardiovascular disease: current evidence, basic mechanisms and research priorities. *European Respiratory Journal*.

[55] Mehra R, Benjamin EJ, Shahar E (2006). Association of nocturnal arrhythmias with sleep-disordered breathing: the sleep heart health study. *American Journal of Respiratory and Critical Care Medicine*.

[56] Hoffstein V, Mateika S (1994). Cardiac arrhythmias, snoring, and sleep apnea. *Chest*.

[57] Cicek D, Lakadamyali H, Gokay S, Sapmaz I, Muderrisoglu H (2012). Effect of obstructive sleep apnea on heart rate, heart rate recovery and QTc and P-wave dispersion in newly diagnosed untreated patients. *American Journal of the Medical Sciences*.

[58] Dursunoglu D, Dursunoglu N (2007). Effect of CPAP on QT interval dispersion in obstructive sleep apnea patients without hypertension. *Sleep Medicine*.

[59] Gami AS, Howard DE, Olson EJ, Somers VK (2005). Day-night pattern of sudden death in obstructive sleep apnea. *The New England Journal of Medicine*.

[60] Fleetham J, Ayas N, Bradley D (2011). Canadian Thoracic Society 2011 guideline update: diagnosis and treatment of sleep disordered breathing. *Canadian Respiratory Journal*.

[61] Abe H, Takahashi M, Yaegashi H (2010). Efficacy of continuous positive airway pressure on arrhythmias in obstructive sleep apnea patients. *Heart and Vessels*.

[62] Patel D, Mohanty P, Di Biase L (2010). Safety and efficacy of pulmonary vein antral isolation in patients with obstructive sleep apnea: the impact of continuous positive airway pressure. *Circulation*.

[63] Naruse Y, Tada H, Satoh M (2013). Concomitant obstructive sleep apnea increases the recurrence of atrial fibrillation following radiofrequency catheter ablation of atrial fibrillation: clinical impact of continuous positive airway pressure therapy. *Heart Rhythm*.

[64] Bazan V, Grau N, Valles E Obstructive sleep apnea in patients with typical atrial flutter: prevalence and impact on arrhythmia control outcome.

[65] Murri M, Alcázar-Ramírez J, Garrido-Sánchez L (2009). Oxidative stress and metabolic changes after continuous positive airway pressure treatment according to previous metabolic disorders in sleep apnea-hypopnea syndrome patients. *Translational Research*.

[66] Ishida K, Kimura F, Imamaki M (2006). Relation of inflammatory cytokines to atrial fibrillation after off-pump coronary artery bypass grafting. *European Journal of Cardio-Thoracic Surgery*.

[67] Deftereos S, Giannopoulos G, Kossyvakis C (2012). Colchicine for prevention of early atrial fibrillation recurrence after pulmonary vein isolation: a randomized controlled study. *Journal of the American College of Cardiology*.

[68] Dziewas R, Imai T, Dittrich R (2006). Night-time bradyarrhythmia in a patient with mild obstructive sleep apnea syndrome is reversed with CPAP treatment. *Journal of Clinical Sleep Medicine*.

[69] Koehler U, Fus E, Grimm W (1998). Heart block in patients with obstructive sleep apnoea: pathogenetic factors and effects of treatment. *European Respiratory Journal*.

[70] Abdelghani A, Slama S, Hayouni A (2009). Acceptance and long-term compliance to continuous positive airway pressure in obstructive sleep apnea. A prospective study on 72patients treated between 2004 and 2007. *Revue de Pneumologie Clinique*.

[71] Rolfe I, Olson LG, Saunders NA (1991). Long-term acceptance of continuous positive airway pressure in obstructive sleep apnea. *American Review of Respiratory Disease*.

[72] Sopkova Z, Dorkova Z, Tkacova R (2009). Predictors of compliance with continuous positive airway pressure treatment in patients with obstructive sleep apnea and metabolic syndrome. *Wiener Klinische Wochenschrift*.

